# First Diagnosis of Gitelman Syndrome During Pregnancy in an Adolescent Female: A Case Report

**DOI:** 10.7759/cureus.59644

**Published:** 2024-05-04

**Authors:** Konstantinos Zacharis, Chalent Alexakis, Vasiliki K Tsapadikou, Ismini Anagnostaki, Theodoros Charitos

**Affiliations:** 1 Department of Obstetrics and Gynaecology, General Hospital of Lamia, Lamia, GRC

**Keywords:** hypomagnesemia, adolescent pregnancy, metabolic alkalosis, hypokalemia, gitelman syndrome

## Abstract

Gitelman syndrome (GS) is an inherited somatic recessive disorder characterized by hypokalemic metabolic alkalosis, accompanied by hypocalciuria and hypermagnesuria. It usually presents in late childhood or young adults with muscle weakness, tetany, or convulsions. Limited information is available in the literature regarding the proper management of this syndrome during pregnancy, as well as its effects on both the mother and the child. We herein present the case of a 16-year-old primigravida who was admitted to the emergency department with chief complaints of abdominal pain, weakness, and vomiting for the past three days during the 12th week of gestation. Routine blood investigations revealed hypokalemia and hypomagnesemia, and electrocardiography (ECG) showed ST-segment depressions. Further evaluation was performed due to persistent hypokalemia, and metabolic alkalosis, hypocalciuria, and hyperaldosteronism were found. Hence, a clinical diagnosis of GS took place. The pregnancy progressed smoothly without complications; potassium levels remained consistently below normal, requiring supplementation three times during pregnancy. Pregnant women with GS should be reported due to the rarity of cases, aiming to establish a standardized approach for monitoring and management.

## Introduction

Gitelman syndrome (GS) was identified as a distinct clinical condition in 1966 by Gitelman et al. [1] after previously being classified under Bartter syndrome. This syndrome represents a rare autosomal recessive kidney disorder, stemming from a genetic mutation in the *SLC12A3* gene, responsible for encoding the thiazide-sensitive sodium-chloride cotransporter in the distal convoluted tubule [2]. This mutation is associated with familial hypokalemia-hypomagnesemia, characterized by metabolic alkalosis, significant hypomagnesemia, and reduced calcium excretion in urine.

With an estimated prevalence of one in 40,000, GS also holds a 1% heterozygote prevalence in Caucasian populations, making it one of the more common inherited renal tubular disorders [2]. Typically, symptoms do not manifest before the age of six, with diagnosis typically occurring during adolescence or adulthood. Common symptoms among GS patients include intermittent episodes of muscle weakness and tetany, sometimes accompanied by abdominal pain, vomiting, and fever [3].

Hypokalemia during pregnancy can arise as a secondary condition due to the physiological adaptations that occur during this period, or it may result from other common causes such as hemodilution, hyperemesis gravidarum, and diarrhea. However, a hidden and less common cause may be GS [4]. We hereby present a case of an adolescent female with a first diagnosis of GS during pregnancy.

## Case presentation

**Figure 1:**
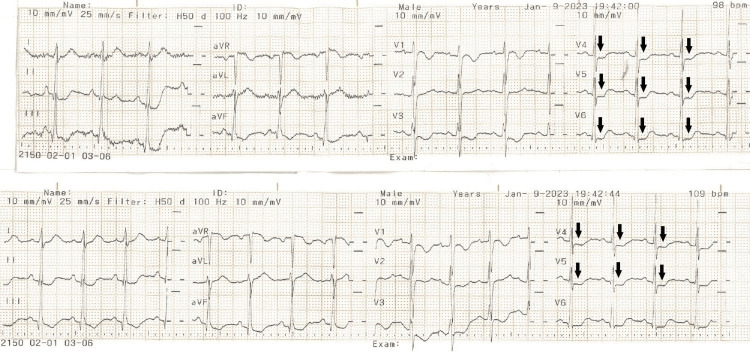
ECG of the adolescent female during admission showing ST-segment depressions (black arrows) ECG: electrocardiogram

A 16-year-old, G1P0 patient presented at 12 weeks of gestation due to abdominal pain, weakness, nausea, and vomiting for the past three days. Upon examination, hypokalemia (2.8 mEq/L) and hypomagnesemia (0.9 mg/dL) were detected, along with ST-segment disturbances on the electrocardiogram (ECG), consistent with hypokalemia (Figure 1).

Further investigation was conducted due to persistent hypokalemia, revealing hypocalciuria, metabolic alkalosis, and hyperaldosteronism (serum aldosterone in the upright position: 66 ng/dL), leading to the patient being referred to a tertiary hospital for pediatric nephrological evaluation. Clinical diagnosis of Gitelman syndrome was established, and antenatal monitoring of the patient continued at our clinic's outpatient department.

**Figure 2:**
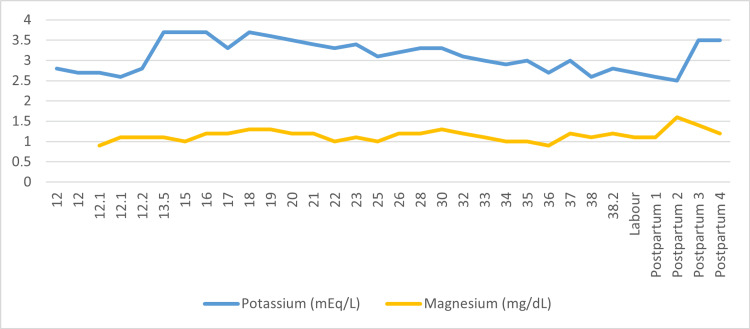
Patient's potassium and magnesium levels throughout pregnancy The horizontal axis represents the weeks of gestation until labor and the postpartum period.

The pregnancy progressed smoothly without complications, and electrolyte levels were measured weekly throughout its duration. Potassium levels remained consistently below normal, ranging from 2.6 to 3.7 mmol/L. Potassium supplementation was required three times via intravenous (IV) administration at gestational ages 33^+6^, 35^+6^, and 37^+6^ weeks (Figure 2). In particular, the patient received IV repletion, consisting of 3 g of potassium chloride and 2.5 g of magnesium sulfate infused in 1,000 mL of dextrose 5% in water over six hours, on each occasion.

At 38^+4^ weeks of gestation, she gave birth to a male infant weighing 3,170 g via assisted vaginal delivery due to failure to progress. During labor, potassium levels further decreased, necessitating correction through intravenous potassium administration. She was discharged with instructions for regular nephrological follow-up and systematic intake of potassium and magnesium supplements.

## Discussion

The exact frequency of GS is not known. It is transmitted in an autosomal recessive manner, although cases with autosomal dominant inheritance have also been reported [5]. Various theories have been proposed regarding the pathophysiology of GS, such as inadequate response to angiotensin II, overproduction of natriuretic factor, obligatory renal potassium loss, and disturbances in prostaglandin metabolism [6]. However, since the 1970s, the most accepted hypothesis, supported by molecular biology methods, is the reduction of sodium-chloride transporters in the distal convoluted tubule, caused by mutations in the gene encoding proteins crucial for the reabsorption of sodium and chloride. Recent studies have shown mutations in the *SLC12A3* gene in the majority of GS patients. This gene, located on chromosome 16q13, contains 26 exons and encodes the renal cotransporters of sodium chloride sensitive to thiazide, primarily located in the distal convoluted tubule [2]. These receptors are known internationally by various abbreviations: TSC, NCC, NCCT, and ENCC1. More than 100 gene mutations have been described in GS patients [7].

GS patients may be asymptomatic in the first years of life [8]. The first clinical manifestation usually occurs in childhood or adolescence. The most common symptoms include salt craving, cramps primarily affecting the upper and lower extremities and occurring more frequently at night or during moderate physical activity, and general weakness. Severe cases may present with muscle paralysis and rhabdomyolysis [8]. Additionally, joint pain, especially in the fingers or knees, dizziness, paresthesia, tetany, palpitations, and difficulty concentrating are often reported. Patients frequently experience polyuria and nocturia. Delayed growth in GS patients is usually mild. Their blood pressure is normal and even lower than that of unaffected relatives, despite the increased salt intake they usually have in their diet. It is not clear whether this is due to chronic hypovolemia and sodium loss or to secondary resistance to circulating factors causing hypertension. Prolongation of the QTc interval on the electrocardiography due to potassium and magnesium disturbances predisposes to dangerous arrhythmias [8]. GS can be easily misdiagnosed or overlooked due to its low incidence, nonspecific symptoms, and lack of awareness [9].

During pregnancy, numerous physiological and metabolic changes occur. Renal blood flow increases, primarily due to elevated cardiac output and vasodilation of the vessels in the kidneys. By the end of the first trimester, glomerular filtration rate (GFR) undergoes a significant 70% increase (reaching approximately 150 mL/minute), attributed to the augmented renal blood flow (rising by 55%-80%). This elevation in GFR persists until the 36th week to uphold a physiological electrolyte balance [10]. In GS, this delicate equilibrium is disrupted by a tubular defect, leading to uncontrolled loss of potassium and magnesium. Consequently, there is a heightened need for external supplementation, as observed in our patient. The objective of therapy for pregnant women with GS is to achieve serum levels of potassium and magnesium that ensure patients remain asymptomatic and promote a successful perinatal outcome [11]. The only adverse perinatal effect mentioned in the literature is oligohydramnios, which is associated with the use of potassium-sparing diuretics but with no effect in the neonate [4].

## Conclusions

The experience of clinical physicians in managing pregnant women with GS is limited. There is no evidence of serious risk to the embryo, but the mother's symptoms may worsen during pregnancy. Because cases of pregnant women with GS are uncommon and rarely reported, there are no established guidelines for the perinatal care of women with the disease. According to the studies published so far and our own case, pregnant women with GS may have an uncomplicated pregnancy, provided there is close monitoring, regular evaluation of electrolytes, and adequate supplementation with potassium and magnesium supplements. The approach to women with GS requires an interdisciplinary team, aiming for a smooth perinatal outcome for both the mother and the newborn.
